# Maxillofacial trauma patient: coping with the difficult airway

**DOI:** 10.1186/1749-7922-4-21

**Published:** 2009-05-27

**Authors:** Amir A Krausz, Imad Abu el-Naaj, Michal Barak

**Affiliations:** 1 Department of Oral and Maxillofacial Surgery, Rambam Health Care Campus, Haifa, Israel; 2 Department of Anesthesiology, Rambam Health Care Campus, and Bruce Rappaport Faculty of Medicine, Technion-Israel Institute of Technology, Haifa, Israel

## Abstract

Establishing a secure airway in a trauma patient is one of the primary essentials of treatment. Any flaw in airway management may lead to grave morbidity and mortality. Maxillofacial trauma presents a complex problem with regard to the patient's airway. By definition, the injury compromises the patient's airway and it is, therefore, must be protected. In most cases, the patient undergoes surgery for maxillofacial trauma or for other, more severe, life-threatening injuries, and securing the airway is the first step in the introduction of general anaesthesia. In such patients, we anticipate difficult endotracheal intubation and, often, also difficult mask ventilation. In addition, the patient is usually regarded as having a "full stomach" and has not been cleared of a C-spine injury, which may complicate airway management furthermore. The time available to accomplish the task is short and the patient's condition may deteriorate rapidly. Both decision-making and performance are impaired in such circumstances. In this review, we discuss the complexity of the situation and present a treatment approach.

## Introduction

The first priority in assessing and managing the trauma patient is airway maintenance with cervical spine control. This is based on the Advanced Trauma Life Support (ATLS) concept for managing patients who sustained life-threatening injuries [1]. According to that concept, loss of an airway kills more quickly than does the loss of the ability to breathe or circulatory problems. Thus, life saving intervention should begin with airway management, when required [1,2]. Indeed, problems in airway management could lead to grave morbidity and mortality in the general surgical population [3,4] as well as in trauma patients [5].

Airway management problems are not confined to the early stages of 'triage' or to the resuscitation of the patient. Morbidity and mortality of in-hospital trauma patients often result from critical care errors. The most common critical care errors are related to airway and respiratory management [5,6]. Gruen et al studied 2594 trauma mortality patients in order to identify patterns of errors contributing to inpatient deaths [6]. They found that failure to intubate, secure or protect the airway was the most common factor related to patient mortality, responsible for 16% of inpatient deaths.

### Maxillofacial Trauma and Airway Injuries

Immediate management of maxillofacial injuries is required mainly when impending or existing upper airway compromise and/or profuse hemorrhage occurs. Hutchinson et al [7] addressed six specific situations associated with maxillofacial trauma, which may adversely affect the airway:

1. Posteroinferior displacement of a fractured maxilla parallel to the inclined plane of the skull base may block the nasopharyngeal airway.

2. A bilateral fracture of the anterior mandible may cause the fractured symphysis to slide posteriorly along with the tongue attached to it via its anterior insertion. In the supine patient, the base of the tongue may drop back, thus blocking the oropharynx.

3. Fractured or exfoliated teeth, bone fragments, vomitus and blood as well as foreign bodies – dentures, debris, shrapnel etc. – may block the airway anywhere along the upper aerodigestive tract.

4. Hemorrhage, either from distinct vessels in open wounds or severe nasal bleeding from complex blood supply of the nose, may also contribute to airway obstruction.

These situations should be addressed immediately using various manual and/or instrumental techniques, in accordance with the "A" step in the ABC treatment protocol suggested by the ATLS [1]. Endotracheal intubation should be considered if it was not performed earlier.

5. Soft tissue swelling and edema resulting from trauma to the head and neck may cause delayed airway compromise.

6. Trauma to the larynx and trachea may cause swelling and displacement of structures, such as the epiglottis, arytenoid cartilages and vocal cords, thereby increasing the risk of cervical airway obstruction.

A high index of suspicion, meticulous physical examination and close observation of the patient may assist in the early detection of such situations and facilitate proper and timely management in order to avoid future complications.

Once airway management has been completed and all hemorrhage sites controlled, definitive management of bone and soft tissue injuries resulting from maxillofacial trauma may be deferred until life- and/or organ-threatening injuries have been properly managed.

### The Complexity of the situation

The maxillofacial trauma patient often presents a problem of difficult mask ventilation and difficult intubation. The trauma usually disrupts the normal anatomy and causes oedema and bleeding in the oral cavity. The mask cannot be properly close-fitted to the face, to enable effective mask ventilation. Furthermore, an injured airway may prevent efficient air transferring from the musk to the lungs. The challenge in performing the intubation arises mainly from a difficulty in visualizing the vocal cords with conventional direct laryngoscopy. The oral cavity, pharynx and larynx may be filled with blood, secretions, debris, soft tissue and bone fractures, all of which preclude good visualization of the vocal cords.

Apart from the problem of anticipated difficult airway, several other factors may worsen the scenario:

#### C-spine Injury

A patient who sustained supra-clavicular trauma is considered to have a C-spine injury until proven otherwise. Complete C-spine clearance may take hours and sometimes days, and until then the patient's neck must be supported by a collar and all neck movements should be avoided. At the time of intubation the assistant performs "in-line stabilization", in order to support the head and neck in place and prevent neck flexion throughout the procedure [8]. Recent data indicate, on one hand, that direct laryngoscopy and intubation are unlikely to cause clinically significant neck movements and, on the other hand, "in-line stabilization" may not always immobilize injured segments effectively. In addition, manual "in-line stabilization" degrades the laryngoscopic view which may, in turn, cause hypoxia and worsen the outcome in traumatic brain injury [9,10]. Another approach suggested by Robitaille et al. is to use the GlideScope videolaryngoscopy for intubation rather than the commonly used Macintosh blade, thus minimizing neck movements [11].

#### Full stomach

The maxillofacial trauma patient, as every trauma patient, is considered to have a "full stomach", since there was no time for stomach emptying prior to intubation. In addition, this patient often bleeds from the upper aerodigestive tract: blood is swallowed and accumulates in the stomach, and the risk of regurgitation and aspiration is high. In order to diminish such risks, evacuating the contents of the stomach through the naso-gastric tube before proceeding with airway management is recommended. However, insertion of a naso-gastric tube in a confused, uncooperative, sometimes intoxicated patient who sustained a facial injury may, by itself, trigger vomiting. Another means of reducing the risk of aspiration is to use Sellick's manoeuvre [12]. Sellick described a technique in which pressure is applied to the cricoid cartilage, thereby compressing the oesophagus against the underlying vertebral body. The pathway of regurgitated gastric contents into the mouth is occluded and aspiration is prevented. Over the years Sellick's manoeuvre, or cricoid pressure, has been incorporated into an overall approach referred to as 'rapid sequence induction', intended to minimize the risk of aspiration. Although cricoid pressure and rapid sequence induction are widely used, the effectiveness and safety of the technique have been questioned [13]. Several studies have shown that cricoid pressure may significantly worsen the laryngeal view, making endotracheal intubation even more difficult [14-16].

#### Emergency Situations

Managing the airway in an emergent situation poses additional difficulty, resulting from the fact that the time to accomplish the task is short and the patient's condition may deteriorate quickly. Both decision-taking and performance are impaired at such times. The performance of urgent or emergent intubation is associated with remarkably high complication rates, which may exceed 20% [17-20]. This is the result of several factors, including repeated intubation attempts, performing direct laryngoscopy without muscle relaxation and lack of operator experience.

#### Personnel Experience

After facing the complexity of managing the maxillofacial injured patient and deciding on treatment priorities, execution of the treatment plan should commence. The advantage of skillful, experienced personnel has been established in several studies. Schmidt et al prospectively investigated emergent tracheal intubatuions [21] and found that supervision by an Attending Anesthesiologist was associated with a decreased incidence of complications. Hodzovic et al studied fibreoptic intubation in a manikin using three airway conduits, and found that Senior House Officers were significantly slower than both Specialist Registrars and Consultants in achieving the goal [22]. However, in emergency situations, the caretakers are often the less experienced. This is the "inverse care law", meaning that the care for those who are most critically ill is provided by those who are not- yet the most expert [23]. In the same way the responsibility for acute airway management often falls into the hands of non-anesthesiologists [24]. This may be futile if not risky or disastrous for the maxillofacial trauma patient. Within time and place limitations, we believe that the most experienced personnel should perform the difficult task of airway management in traumatized patients.

### Approach to the Maxillofacial Trauma Patient's Airway Management

#### Airway Evaluation and Preparation

Airway evaluation should be as thorough and as quick as possible, due to the fact that the patient's airway is compromised. Nevertheless, defining the exact difficulty involved could direct the physician to the best approach to managing that airway.

**Figure 1:**
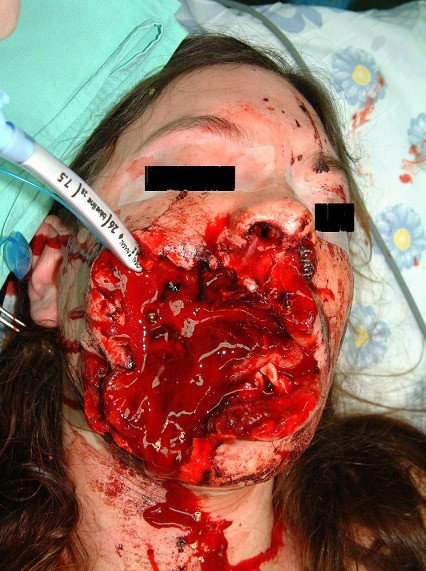
**A woman who sustained a single gunshot injury**. She arrived at the hospital conscious and breathing spontaneously. Impossible mask ventilation and diffucult intubation were anticipated. Direct laryngoscopy was performed and oro-tracheal intubation was successful.

**Figure 2:**
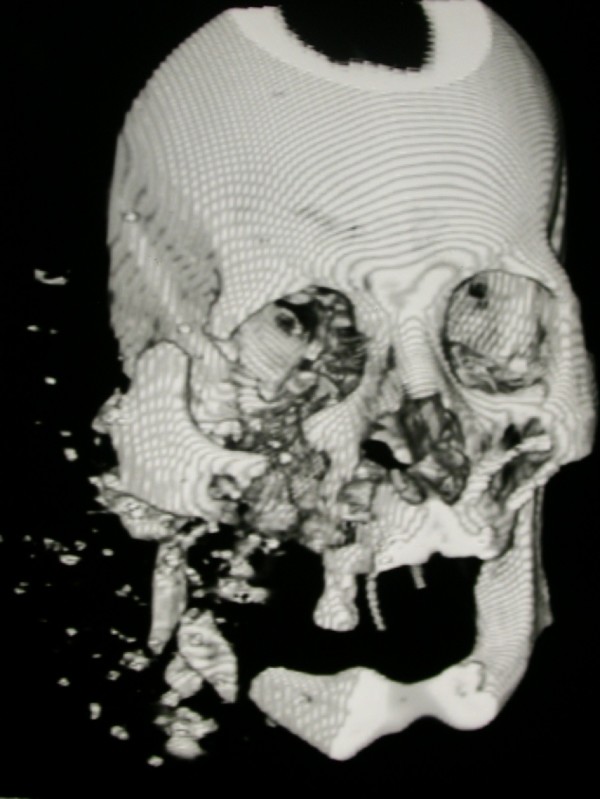
**A patient with high velocity long distance injury, with severe soft tissue damage of the right chick**. 3 dimensions CT shows comminuted fracture of the right orbit, zygoma and right mandible.

**Figure 3:**
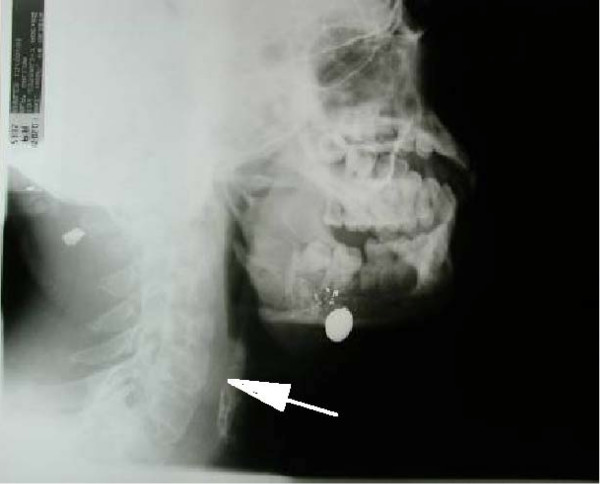
**Male patient who sustained low velocity missile injury to the left chick**. Lateral radiography demonstrates the bullet location. Note the patent airway on the lateral view (white arrow).

**Figure 4:**
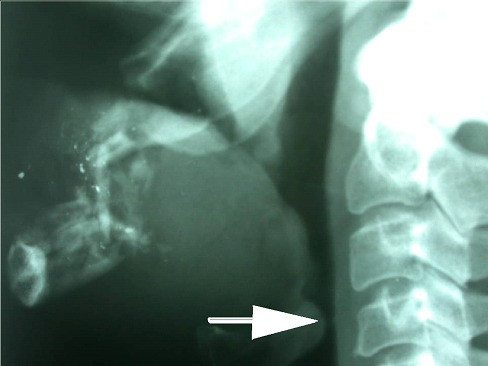
**Male patient who sustained high velocity injury to the lower face**. Tracheostomy was performed in the Shock-Trauma Unit. Lateral x-ray shows comminuted fracture of the mandible with huge soft tissue swelling of the neck and narrowing of the airway (white arrow).

The questions that should be answered are:

• Is the patient conscious? If so, the use of sedation or analgesics should be done cautiously since the airway can be lost following injudicious use of such drugs [25].

• Is he/she breathing spontaneously? If so, there is time to arrive at the hospital, preferably to the operating room, and manage the airway under the best conditions and by the most experienced personnel. Failed attempts at endotracheal intubation by non-qualified caretakers could cause rapid deterioration. Indeed, according to the American Society of Anesthesiologists (ASA) Practice Guidelines for management of the difficult airway, spontaneous breathing should be preserved in patients with anticipated difficult endotracheal intubation [26].

• What is the extent, the composition and the anatomy of the injury? Figure 1 shows patient with very extensive injury to the face, where mask ventilation was not possible and tracheal intubation was very difficult (Figure 1).

• How extensive is the damage to the bony structures of the face? In cases of massive injuries, mask ventilation may be impossible, while injury limited to the soft tissues may enable mask ventilation. Figure 2 shows 3 dimensions CT of a patient with comminuted fracture of the right orbit, zygoma and right mandible.

• Is there a limitation in mouth opening? Is that limitation the result of pain and after sedation the mouth could be opened wider? The answer for this question depends, among other things, on the clinical and radiological evidence of a temporo-mandibular joint (TMJ) injury. If the limitation in mouth opening is caused by a TMJ injury, sedation will not improve mouth opening, will not help in managing the airway, and may worsen the scenario.

• Is there soft tissue oedema and pressure on the airway? Figure 3 shows lateral radiography of a patient who sustained low velocity missile injury to the left chick. The radiograph demonstrates the bullet location and the patent airway. Figure 4 is the lateral x-ray of a patient with comminuted fracture of the mandible with huge soft tissue swelling of the neck and narrowing of the airway.

As with every difficult airway situation, the staff and equipment for difficult intubation should be prepared and ready to use. The approach should be chosen according to the patient's injuries, airway status and the care provider's experience with such equipment and procedures.

#### Treatment Options

As stated earlier, the challenge in performing endo-tracheal intubation arises mainly from the difficulty in visualizing the vocal cords. Numerous airway devices and equipment have been developed to overcome this obstacle [27]. Some, such as the fiberoptic bronchoscope, enable indirect visualization of the vocal cords. Others, such as the laryngeal mask airway (LMA) or Combitube (esophageal-tracheal twin-lumen airway device), are inserted blindly and do not require visualization of the vocal cords by any means [28]. The final option is creating a surgical airway via cricithyrotomy or tracheotomy, thus bypassing the larynx and establishing direct access to the trachea.

The scope of this review is limited and therefore we chose to focus on several principle airway devices and describe their suitability for the trauma patient.

### Indirect visualization of the vocal cords

**Flexible fiberoptic intubation **under local anaesthesia is the technique of choice for management of the anticipated difficult intubation and difficult mask ventilation in the patient undergoing an elective procedure [26]. The option of fiberoptic intubation is suitable for elective procedures but impractical in maxillofacial trauma patients. Blood, vomitus and secretions in the patient's airway preclude vision by fiberoptic instruments. In addition, accomplishing effective local anesthesia in the traumatized region is difficult. Furthermore, the patient's cooperation is essential for such an approach, but not always possible in the traumatized patient.

**GlideScope **is a video laryngoscope which enables indirect visualization of the epiglottis. Like many other indirect fiberoptic and video-based instruments, it was developed as a potential alternative to direct laryngoscopy for cases involving difficult intubation [29].

However, all these instruments rely on good vision of the inner airway, which is precluded in the trauma patient by blood and secretions. From this point of view, those instruments are not more advantageous than the fiberoptic bronchoscope.

### Blind Airway Devices

**Laryngeal mask airway (LMA) **is one of the most important developments in airway management devices. It is inserted blindly and requires minimal experience. However, the LMA does not provide a definitive airway; it is a supraglotic ventilatory device and, as such, may cause stomach inflation and may be displaced when the patient is moved and managed. Thus, it is not suitable for managing trauma patients. However, it could enable ventilating the patient until definitive airway is achieved, functioning in bridging the period of early treatment.

**Combitube (esophageal-tracheal twin-lumen airway device) **is inserted blindly. Yet, tissue damage and disruption of the anatomy increase the risk of false route and further damage to the airway. Furthermore, Combitube insertion is associated with serious complications to the upper aerodigestive tract, as was demonstrated with its use in the pre-hospital setting, such as esophageal laceration and perforation, tongue oedema, vocal cord injury, tracheal injury, aspiration pneumonitis and pneumomediastinum [30].

### Surgical Airway

Performing a cricothyrotomy or tracheotomy under local anaesthesia is a relatively safe option for managing the airway [31]. However, this approach has its drawbacks. This procedure could be uncomfortable or even painful for the patient, who is already experiencing severe pain and emotional stress. Tracheotomy by itself carries a 5% risk of complications, such as haemorrhage or pneumothorax [32]. Nevertheless, if the maxillofacial trauma is extensive and requires maxillo-mandibular fixation for several weeks or if prolonged mechanical ventilation is probable, surgical airway may be the best option in such cases. The surgical approach is also used as an emergency salvage procedure, when other options have failed [33].

### Direct Laryngoscopy

Last but not least lies the classic approach of direct laryngoscopy. This simple and straightforward approach to the airway may be successful in the hands of experienced personnel, though the risk of losing grip on the airway is high. Thus, this approach should be reserved for selected slim patients with good surface anatomy of the neck, where urgent cricothyrotomy or tracheotomy is feasible, and when an ENT specialist is ready to perform.

### Post-operative Management

The patient with a difficult airway is also at high risk for complications in the post-operative period. Following surgery, mucous membranes are oedematous, soft tissue is swollen and the air pathway may be compressed. Neck expandability is relatively low and even a small haemorrhage in the region could result in airway compromise. The risk of airway-related complications during the peri-operative period was studied by Peterson et al [4]. They analyzed the American Society of Anesthesiologists Closed Claims database to identify the patterns of liability associated with the management of the difficult airway. They found that complications arose throughout the peri-operative period: 67% upon induction, 15% during surgery, 12% at extubation, and 5% during recovery.

In intubated maxillofacial trauma patients, extubation should be deferred until normal anatomy is restored or at least until the oedema subsides. During extubation the patient should be monitored closely and the care providers should be prepared for the possibility of re-intubation. In a case of tracheotomy tube, the patient may be awakened and allowed to breathe spontaneously through the tracheostomy tube for a few days, providing a safer recovery.

## Conclusion

Airway management of the maxillofacial trauma patient is complex and requires both sound judgement and considerable experience, which are gained in similar emergency situations. Skilful and experienced personnel are mandatory, as is collaboration by the anesthesiologist, maxillofacial surgeon, ENT specialist or general surgeon, in order to have an outcome with minimal risks and maximal success. It is important to remember that timely, decisive and skillful management of the airway can often make the difference between life and death or between ability and disability in such situations.

## Consent

Written informed consent was obtained from the patient for publication of the publication of their case reports and accompanying images. A copy of the written consent is available for review by the Editor-in-Chief of this journal.

## Competing interests

The authors declare that they have no competing interests.

## Authors' contributions

The review is the product of the collaboration of AAK, IA and MB, each one contributed of his/her knowledge and expertise. All authors read and approved the final manuscript.

## References

[B1] American College of Surgeons Committee on Trauma (2004). Advanced Trauma Life Support for Doctors ATLS.

[B2] Walls RM (1998). Management of the difficult airway in the trauma patient. Emerg Med Clin North Am.

[B3] Domino KB, Posner KL, Caplan RA, Cheney FW (1999). Airway injury during anesthesia: a closed claims analysis. Anesthesiology.

[B4] Peterson GN, Domino KB, Caplan RA, Posner KL, Lee LA, Cheney FW (2005). Management of the difficult airway: a closed claims analysis. Anesthesiology.

[B5] Garcia A (2006). Critical care issues in the early management of severe trauma. Surg Clin North Am.

[B6] Gruen RL, Jurkovich GJ, McIntyre LK, Foy HM, Maier RV (2006). Patterns of errors contributing to trauma mortality: lessons learned from 2,594 deaths. Ann Surg.

[B7] Hutchison I, Lawlor M, Skinner D (1990). ABC of major trauma. Major maxillofacial injuries. BMJ.

[B8] Crosby ET (2006). Airway management in adults after cervical spine trauma. Anesthesiology.

[B9] Manoach S, Paladino L (2007). Manual in-line stabilization for acute airway management of suspected cervical spine injury: historical review and current questions. Ann Emerg Med.

[B10] Santoni BG, Hindman BJ, Puttlitz CM, Weeks JB, Johnson N, Maktabi MA, Todd MM (2009). Manual in-line stabilization increases pressures applied by the laryngoscope blade during direct laryngoscopy and orotracheal intubation. Anesthesiology.

[B11] Robitaille A, Williams SR, Tremblay MH, Guilbert F, Thériault M, Drolet P (2008). Cervical spine motion during tracheal intubation with manual in-line stabilization: direct laryngoscopy versus GlideScope videolaryngoscopy. Anesth Analg.

[B12] Sellick BA (1961). Cricoid pressure to control regurgitation of stomach contents during induction of anaesthesia. Lancet.

[B13] Ellis DY, Harris T, Zideman D (2007). Cricoid pressure in emergency department rapid sequence tracheal intubations: a risk-benefit analysis. Ann Emerg Med.

[B14] Levitan RM, Kinkle WC, Levin WJ, Everett WW (2006). Laryngeal view during laryngoscopy: a randomized trial comparing cricoid pressure, backward-upward-rightward pressure, and bimanual laryngoscopy. Ann Emerg Med.

[B15] Noguchi T, Koga K, Shiga Y, Shigematsu A (2003). The gum elastic bougie eases tracheal intubation while applying cricoid pressure compared to a stylet. Can J Anaesth.

[B16] Haslam N, Parker L, Duggan JE (2005). Effect of cricoid pressure on the view at laryngoscopy. Anaesthesia.

[B17] Mort TC (2007). Complications of emergency tracheal intubation: immediate airway-related consequences: part II. J Intensive Care Med.

[B18] Li J, Murphy-Lavoie H, Bugas C, Martinez J, Preston C (1999). Complications of emergency intubation with and without paralysis. Am J Emerg Med.

[B19] Benedetto WJ, Hess DR, Gettings E, Bigatello LM, Toon H, Hurford WE, Schmidt U (2007). Urgent tracheal intubation in general hospital units: an observational study. J Clin Anesth.

[B20] Mort TC (2004). Emergency tracheal intubation: complications associated with repeated laryngoscopic attempts. Anesth Analg.

[B21] Schmidt UH, Kumwilaisak K, Bittner E, George E, Hess D (2008). Effects of supervision by attending anesthesiologists on complications of emergency tracheal intubation. Anesthesiology.

[B22] Hodzovic I, Petterson J, Wilkes AR, Latto IP (2007). Fibreoptic intubation using three airway conduits in a manikin: the effect of operator experience. Anaesthesia.

[B23] Boylan JF, Kavanagh BP (2008). Emergency airway management: competence versus expertise?. Anesthesiology.

[B24] Kovacs G, Law JA, Ross J, Tallon J, MacQuarrie K, Petrie D, Campbell S, Soder C (2004). Acute airway management in the emergency department by non-anesthesiologists. Can J Anaesth.

[B25] Peralta R, Hurford WE (2000). Airway trauma. Int Anesthesiol Clin.

[B26] American Society of Anesthesiologists Task Force on Management of the Difficult Airway (2003). Practice guidelines for management of the difficult airway: an updated report by the American Society of Anesthesiologists Task Force on Management of the Difficult Airway. Anesthesiology.

[B27] Hagberg C, Lam N, Brambrink A (2007). Current concepts in airway management in the operating room: A new approach to the management of both complicated and uncomplicated airways. Curr Rev Clin Anesth.

[B28] Rabitsch W, Schellongowski P, Staudinger T, Hofbauer R, Dufek V, Eder B, Raab H, Thell R, Schuster E, Frass M (2003). Comparison of a conventional tracheal airway with the Combitube in an urban emergency medical services system run by physicians. Resuscitation.

[B29] Koerner IP, Brambrink AM (2005). Fiberoptic techniques. Best Pract Res Clin Anaesthesiol.

[B30] Vézina MC, Trépanier CA, Nicole PC, Lessard MR (2007). Complications associated with the Esophageal-Tracheal Combitube in the pre-hospital setting. Can J Anaesth.

[B31] Helm M, Gries A, Mutzbauer T (2005). Surgical approach in difficult airway management. Best Pract Res Clin Anaesthesiol.

[B32] Kearney PA, Griffen MM, Ochoa JB, Boulanger BR, Tseui BJ, Mentzer RM (2000). A single-center 8-year experience with percutaneous dilational tracheostomy. Ann Surg.

[B33] Dob DP, McLure HA, Soni N (1998). Failed intubation and emergency percutaneous tracheostomy. Anaesthesia.

